# A283 ROLE OF IMMUNOGLOBULIN E IN ABDOMINAL PAIN DUE TO INTERACTIONS BETWEEN DIET AND STRESS IN A MODEL OF IBS

**DOI:** 10.1093/jcag/gwac036.283

**Published:** 2023-03-07

**Authors:** C Lopez Lopez, J Jaramillo Polanco, Q K Tsang, A E Lomax, S J Vanner, D E Reed

**Affiliations:** Gastrointestinal Diseases Research Unit, Queen's University, Kingston, Canada

## Abstract

**Background:**

Food and stress are common triggers of symptoms in IBS patients. We previously showed in a murine model of IBS that exposure to a food antigen during a period of psychological stress leads to visceral hypersensitivity in both the ileum and colon upon re-exposure to the food antigen. This was inhibited by blocking mast cells and histamine receptors, suggesting that mast cell activation is involved in this increased pain signaling. However, it is unknown if immunoglobulin E (IgE) is involved in the activation of mast cells leading to visceral hypersensitivity.

**Purpose:**

Determine the role of IgE in the increased pain signaling in ileum and colon of mice re-exposed to ovalbumin after an initial exposure to ovalbumin during chronic stress.

**Method:**

Balb/c mice underwent water avoidance stress (WAS) for 10 days and were exposed to ovalbumin (OVA) from days 2-10. Five weeks later, mice were re-exposed to ovalbumin for two weeks (WAS/OVA+OVA mice). Mice were injected with anti-mouse IgE or isotype control antibody (1 mg/kg, i.p.) just prior and during ovalbumin re-exposure. *Ex-vivo* extracellular afferent recordings from nerves innervating the ileum were obtained to assess changes in mechanosensitivity. Ileum and colonic supernatants were also collected. Dorsal root ganglion neurons (DRG) from control mice (n = 12) were incubated with ileal and colonic supernatants. The excitability of these neurons was assessed by measuring the rheobase (minimum current required to evoke an action potential; lower rheobase=increased excitability) using perforated patch clamp. Data were analyzed by unpaired *t*-test or two-way ANOVA with Sidak’s *post hoc* test.

**Result(s):**

WAS/OVA+OVA mice treated with anti-IgE antibody had decreased mechanosensitivity of ileal nerves following lumen distention compared to WAS/OVA+OVA mice treated with isotype control antibody (7.7 ± 0.65 vs 12.9 ± 1.04 Hz, p = 0.034, n = 9 and 8 mice, respectively). Ileal supernatants from anti-IgE treated WAS/OVA+OVA mice evoked lower excitability (higher rheobase) in DRG neurons than neurons incubated with supernatants from isotype control treated WAS/OVA+OVA mice (rheobase: 89.2 ± 4.7 vs 71.9 ± 5.1 pA, p= 0.018). Colonic supernatants showed similar effects of the anti-IgE treatment (rheobase: 95.5 ± 4.4 vs 76.7 ± 5.4 pA, p = 0.01).

**Conclusion(s):**

IgE is involved in the increased pain signaling observed in both ileum and colon induced by antigen re-exposure following an initial stress-food antigen interaction. This suggests that IgE-mediated mast cell activation in response to food antigens may be a mechanism of meal-induced pain in IBS patients.

**Please acknowledge all funding agencies by checking the applicable boxes below:**

Other

**Please indicate your source of funding;:**

Department of Medicine, Translational Medicine Grant, Queen's University

**Disclosure of Interest:**

None Declared

